# XGBoost Classification of Epileptic EEG Using Nonlinear Dynamical Features and SHAP

**DOI:** 10.3390/e28080920

**Published:** 2026-08-17

**Authors:** Xiaojie Lu, Hui Lou, Xiaoyang Jin, Bianmei Zhang

**Affiliations:** 1 School of Medical Information, Wannan Medical University, Wuhu 241002, China; luxiaojie06@wnmc.edu.cn (X.L.);; 2 Anhui Province High-Quality Dataset Construction Base for Smart Healthcare, Wuhu 241002, China; 3 Institute of Medical Artificial Intelligence, Wannan Medical University, Wuhu 241002, China

**Keywords:** EEG, nonlinear dynamic, XGBoost, SHAP

## Abstract

To evaluate whether nonlinear descriptors of electroencephalogram (EEG) signals support interpretable XGBoost classification and to determine how analysis window duration affects performance. A secondary analysis of the public Bonn EEG dataset was performed. Nine nonlinear features were extracted from non-overlapping 1, 5, 10, and 20 s windows after an original-recording-level train/validation/test split, and a multiclass XGBoost model was interpreted with class-specific SHAP values. The model achieved 93.3% overall accuracy; the class-specific AUC values were 0.978 for Z/O, 0.978 for N/F, and 0.984 for S. Across the four fixed-split duration conditions, the 1 s condition had the lowest descriptive performance, whereas the 5, 10, and 20 s conditions were broadly comparable; no uniquely optimal duration was established. The nonlinear-feature/XGBoost framework provides interpretable benchmark segment classification evidence. Because EEG is modeled as a stochastic process and the dataset is small and heterogeneous, the SHAP attributions do not establish physiological causality or clinical diagnostic validity.

## 1. Introduction

Clinical EEG evaluation is primarily based on expert visual interpretation of established morphological, spatial, and temporal features, and this remains the clinical reference process [1]. Computational EEG analysis has nevertheless developed over more than two decades, ranging from nonlinear descriptors and conventional classifiers to ensemble and deep learning models [2,3,4,5,6,7]. In this study, machine learning is used as a research tool for quantifying multivariate patterns and exploring model-relevant signal structure rather than as a replacement for expert EEG interpretation. EEG is treated as a stochastic non-stationary process; nonlinear descriptors summarize its statistical and dynamical organization but do not imply that every recording is generated by a deterministic system.

Among ensemble methods, Extreme Gradient Boosting (XGBoost) builds an additive sequence of regularized decision trees and can represent nonlinear interactions among heterogeneous tabular features while controlling model complexity [8]. These properties are well suited to the present nine-feature EEG representation. Previous studies have applied XGBoost to single-channel seizure detection and to ensembles combining time-, frequency-, and nonlinear-domain features [9]. The novelty investigated here is therefore not the first use of machine learning for EEG but the combined analysis of multiple nonlinear descriptors, window duration, and class-specific SHAP attribution under one leakage-controlled benchmark protocol.

However, the aforementioned studies are generally limited by inadequate model interpretability. Consequently, interpretable Artificial Intelligence (AI) methodologies have attracted increasing scholarly and clinical attention. To dismantle the opposition between predictive performance and model transparency, interpretable Artificial Intelligence (XAI) mechanisms based on cooperative game theory, particularly SHAP (SHapley Additive exPlanations), have begun to be integrated into the field of neuroelectrophysiological analysis. Sun et al. [10] utilized SHAP to deconstruct the decision logic of the XGBoost classifier in identifying ictal and interictal states, demonstrating the directionality of contributions from specific nonlinear features; Zuo et al. [11] applied XGBoost and SHAP to the prognostic outcome evaluation of Infantile Epileptic Spasms Syndrome (IESS), achieving the visualization of clinical risk factors; Rehman et al. [12] further proposed a dual-layer explanatory framework combining SHAP global attribution to clarify the physiological mapping of different frequency band powers and nonlinear indicators at the classification boundary.

Dokare et al. [13] integrated SHAP with simulated annealing optimization, achieving a patient-level non-specific detection accuracy of 96.58%. Hasan et al. [14] leveraged SHAP values to identify discriminative EEG channels, thereby enhancing clinicians’ understanding of model decision logic. Nevertheless, the integration of multi-nonlinear feature representations with SHAP-based interpretability frameworks remains underexplored. Specifically, there is a lack of deep deconstruction regarding the degree of importance of specific nonlinear dynamic indicators (such as entropy values and fractal dimensions).

Accordingly, the objective of this study is to determine whether nine nonlinear descriptors of EEG segments can support transparent three-class XGBoost classification on the Bonn benchmark, to evaluate the effect of 1, 5, 10, and 20 s analysis windows, and to use SHAP to characterize the fitted model’s global and local feature attributions without treating those attributions as physiological causality or clinical diagnosis.

## 2. Methods

### 2.1. Study Design, Dataset, and Analysis Workflow

This retrospective computational study used the de-identified, publicly available Bonn University EEG benchmark originally described by Andrzejak et al. [15]; the present authors did not recruit or intervene with human participants. The dataset comprises 500 artifact-screened single-channel segments (100 each in Z, O, N, F, and S), each containing 4097 samples over 23.6 s. Sets Z and O were scalp EEG from five healthy volunteers with eyes open and closed using the international 10–20 system. Sets N, F, and S were intracranial EEG from five patients undergoing presurgical evaluation for pharmacoresistant epilepsy: N and F contain seizure-free recordings from the hippocampal formation contralateral to and within the epileptogenic zone, respectively, whereas S contains ictal activity. The original signals were recorded with a 128-channel amplifier, digitized at 12-bit resolution and 173.61 Hz, band-pass filtered at 0.53–40 Hz (12 dB/octave), and selected after visual inspection for artifacts [15]. These labels therefore represent recording condition, region, and brain state; they are not equivalent to an independent patient-level diagnostic cohort.

The present work independently constructed the analysis pipeline: grouping Z/O as normal, N/F as interictal, and S as ictal; splitting original recordings before segmentation; generating non-overlapping duration-specific windows; extracting and standardizing nine nonlinear features; fitting the multiclass XGBoost model; comparing four window durations; and performing class-specific SHAP analyses. This workflow, rather than the public acquisition itself, constitutes the authors’ contribution to the study design.

### 2.2. Nonlinear Feature Definitions and Implementation

(1) Sample entropy

Sample entropy (SampEn) was proposed by Richman and Moorman in 2000 [16] as an improved algorithm addressing the bias problem existing in approximate entropy. For a time series *x*_1_, …, *x_N_*, we define $X_{m}(i)\;=\;\{x_{i},x_{i+1},\ldots ,\;x_{i+m-1}\}$ for i=1,2, …, N − m. The corresponding (*m* + 1)-dimensional templates use the same *N* − *m* starting indices. We define the distance between two vectors as the Chebyshev distance, i.e., the maximum absolute difference of their corresponding elements. Given a similarity tolerance *r* (typically 0.1 to 0.25 times the standard deviation of the sequence), we count the number of vectors that match $X_{m}(i)$ (excluding self-matches, i.e., j ≠ i), denoted as $N_{m}(i)$. Define:

(1) $$B_{i}^{m}(r)\;=\;\frac{N_{m}(i)}{N\;-\;m\;-\;1}$$

Perform the same operation on the (*m* + 1)-dimensional reconstructed vectors:

(2) $$A_{i}^{m}(r)\;=\;\frac{N_{m+1}(i)}{N\;-\;m\;-\;1}$$

Sample entropy is defined as

(3) $$\mathrm{SampEn}(m,r,N)=-\ln\left(\frac{\sum_{i=1}^{N-m}A_{i}^{m}(r)}{\sum_{i=1}^{N-m}B_{i}^{m}(r)}\right)$$

The smaller the sample entropy value, the stronger the regularity and the lower the complexity of the sequence. Compared with approximate entropy, sample entropy has better consistency and smaller bias, making it particularly suitable for complexity analysis of physiological time series.

(2) Dispersion Entropy

Dispersion entropy (DE) is a measure of time series complexity/irregularity proposed by Mostafa Rostaghi and Hamed Azami in 2016 [17]. It takes into account both amplitude distribution and temporal structure information, features fast computation and strong anti-noise capability, and is widely used in nonlinear analysis of EEG, ECG, mechanical vibration, and other fields. The core idea of dispersion entropy is to quantize the signal amplitude into a finite number of classes, segment all possible subsequence patterns according to the embedding dimension and time delay, and then count the probability distribution of each pattern. The irregularity of the signal is then quantified by Shannon entropy.

Given the original time series $\;x\;=\;\{x_{1},x_{2},\ldots ,\;x_{N}\}$, it is first mapped to the interval [0, 1] by the normal cumulative distribution function:

(4) $$y_{j}\;=\;\frac{1}{\sigma \sqrt{2\pi }}\int_{-\infty }^{x_{j}}e^{-\frac{(t-\mu {)}^{2}}{2\sigma^{2}}}\mathrm{dt}$$

where μ is the mean of the sequence, and σ is the standard deviation of the sequence. Then, y is linearly transformed and rounded to numbers in the range [1, c]:

(5) $$z_{j}^{c}\;=\;\mathrm{round}\;(c\cdot y_{j}+0.5)$$

Construct the embedded vectors according to the embedding dimension m and time delay d:

(6) $$z_{i}^{m,c}\;=\;\{z_{i}^{c},{\;z}_{i+d}^{c},\;\ldots ,{\;z}_{i+(m-1)d}^{c}\}$$

where i = 1,2,…,N− (m − 1)d. Map the embedded vector $z_{i}^{m,c}$ to an *m*-digit *c*-ary dispersion pattern π, count the occurrence of all dispersion patterns, and compute the probability p(π):

(7) $$p(\pi )\;=\;\frac{\mathrm{Number}(\pi )}{N\;-\;(m\;-\;1)d}$$

where *π* is the number of embedded vectors mapped to dispersion pattern *π*. Finally, substitute the probability distribution into the Shannon entropy formula to obtain the dispersion entropy:

(8) $$\mathrm{DE}(X,m,c,d)\;=\;-\sum_{\pi =1}^{c^{m}}p(\pi )\ln(p(\pi ))$$

The Shannon entropy formula is

(9) $$H(X)=-\sum_{i=1}^{n}p(x_{i})\log_{2}p(x_{i})$$

where $p(x_{i})$ is the probability of event $X_{i}$, and $\sum p(x_{i})\;=\;1$.

Because Shannon entropy depends only on the probability distribution and does not consider the temporal order of values, it cannot directly characterize the dynamic structural information contained in a time series and is not friendly to continuous signals (such as EEG). To address these two issues, dispersion entropy uses the cumulative distribution function mapping to adapt to continuous EEG. It uses the embedding dimension m to capture temporal patterns, thereby reflecting the dynamic changes of EEG.

According to the existing literature, m is usually taken as 2 or 3 to better balance efficiency and information extraction capability, preventing too small a value from failing to detect the signal’s dynamic behavior, and too large a value from causing heavy computational burden and inability to observe small changes. The number of classes c is recommended to be an integer around 6; too small a value will cause different amplitudes to be classified into the same class, while too large a value will be sensitive to noise. The time delay d is usually taken as 1 to avoid aliasing and ensure computational efficiency.

(3) Lempel–Ziv Complexity

Lempel–Ziv Complexity (LZC) was proposed by Lempel and Ziv in 1976 [18]. It measures the irregularity and randomness of a sequence by counting the number of new subsequence patterns appearing in the sequence. The core idea is that the complexity of a sequence is inversely proportional to its compressibility; that is, the more repeated patterns a sequence has, the lower its complexity. For a symbolic sequence of length N, after binarization processing, the number of newly appearing substrings Cu is counted by traversal scanning to obtain the basic complexity. To eliminate the influence of sequence length on the complexity value, normalization is performed:

LZC_N = C(*N*) log_2_(*N*)/*N* (10)

Here, C(*N*) denotes the number of distinct subsequence patterns obtained by the Lempel–Ziv parsing procedure, N denotes the symbolic sequence length, and log_2_(*N*)/*N* provides the standard sequence-length correction. When phase-space reconstruction is applied before symbolization, the reconstructed vectors are defined as follows:

(11) $${\vec{x}}_{i}\;=\;(x_{i},x_{i+d},x_{i+2d},\ldots ,x_{i+(m-1)d})$$

In this expression, m denotes the embedding dimension, and d represents the time delay. After the above reconstruction, the effective sequence length is reduced to

(12) $$N_{\mathrm{eff}}=N-(m-1)d$$

When phase-space reconstruction was applied before symbolization, the effective sequence length was used only as the sequence length in the same normalized LZC expression [19]:

LZC_mod = C(*N_eff*) log_2_(*N_eff*)/*N_eff* (13)

In the implemented feature pipeline, LZC was calculated on median-binarized symbolic sequences using the standard length correction shown in Equations (10) and (13); no logarithmic effective-length approximation was applied to the reported computations. In addition to LZC, this study extracts fuzzy entropy, permutation entropy, Renyi entropy, the Hurst exponent, C0 complexity, and Higuchi fractal dimension to form a multidimensional nonlinear feature space for EEG segment classification.

For reproducibility, the Bonn EEG signals were used as provided by the database, which had been band-pass-filtered at acquisition; no additional artifact rejection was applied. Before model fitting, nonlinear features were standardized with z-score normalization using the training set mean and standard deviation only, and the same transformation was applied to validation and test segments. The feature parameters used in this study were as follows SampEn *m* = 2 and *r* = 0.2 SD; FuzzyEn *m* = 2, *r* = 0.2 SD, and fuzzy power *n* = 2; PermEn *m* = 3 and time delay tau = 1; Renyi entropy order alpha = 2; dispersion entropy *m* = 3, *c* = 6, and *d* = 1; Hurst exponent by rescaled-range estimation; C0 complexity using the mean spectral amplitude threshold; Higuchi fractal dimension *kmax* = 8; and LZC after median binarization.

### 2.3. XGBoost Algorithm Model

XGBoost is a regularized gradient boosting decision tree that belongs to the ensemble learning boosting framework. It fits nonlinear relationships by iteratively training multiple decision trees and summing their weighted outputs, making it suitable for classification of EEG nonlinear features. The model takes an additive model as its core structure, where the predicted value is obtained by accumulating the outputs of all trees [20]:

(14) $${\hat{y}}_{i}\;=\;\sum_{k=1}^{K}f_{k}(x_{i})$$

In this expression, K is the number of trees, and $f_{k}\left(x_{i}\right)$ is the output of the K-th tree for sample i. XGBoost employs a second-order Taylor expansion to optimize the objective function, taking into account both loss minimization and model complexity control:

(15) $$\mathrm{Obj}\;=\;\sum_{i=1}^{n}l(y_{i},{\hat{y}}_{i})+\sum_{k=1}^{K}\Omega (f_{k})$$

Here, $l(y_{i},{\hat{y}}_{i})$ is the classification loss function, and $\Omega (f_{k})\;=\;\gamma T\;+\frac{\;1}{2}\lambda \sum_{j=1}^{T}w_{j}^{2}$ is the regularization term, which prevents overfitting by limiting the number of leaf nodes *T* and the weights $w_{j}$. During model training, the optimal leaf weights and split gains are calculated based on the first-order derivative gi and second-order derivative hi. When the gain exceeds the threshold *γ*, node splitting is performed to achieve pre-pruning. Meanwhile, optimization strategies such as column subsampling, row subsampling, and sparsity-aware split are adopted to improve the training efficiency and generalization ability for high-dimensional EEG features.

The experimental split was performed at the original-recording level before segmentation to avoid leakage between training and test data. The 500 original Bonn recordings were first merged into three classes (Z/O normal, N/F interictal, and S ictal) and stratified into training, validation, and test sets at a 70:15:15 ratio with random seed 42. Non-overlapping windows were then generated within each split; the residual samples at the end of each 23.6 s recording were discarded. Thus, the total segment counts for 1, 5, 10, and 20 s were 11,500, 2000, 1000, and 500, respectively, and the corresponding test segment counts were 1725, 300, 150, and 75. The reported XGBoost run used objective = multi:softprob, num_class = 3, eval_metric = mlogloss, max_depth = 6, learning_rate = 0.1, subsample = 1.0, colsample_bytree = 1.0, reg_lambda = 1, reg_alpha = 0, and 200 boosting rounds.

### 2.4. SHAP

Model performance was evaluated using accuracy, macro-precision, macro-specificity, macro-sensitivity, and macro-AUC. SHAP visualizations were used for post-hoc interpretability analysis rather than as classification performance metrics.

SHAP is a model interpretability method based on the Shapley value from cooperative game theory, proposed by Lundberg and Lee in 2017 [21]. For the fitted XGBoost classifier, TreeExplainer was applied with the training data as the background distribution, feature_perturbation = ‘interventional’, and model_output = ‘raw’. Accordingly, for each class and sample, the SHAP values are additive contributions to the class-specific raw model score (margin), not direct additive changes in predicted probability. The feature values displayed in the SHAP plots are z-standardized model inputs calculated using training set means and standard deviations; they are not measurements in the original physical units. SHAP was used for post hoc interpretation and does not resolve dependence or multicollinearity among correlated features.

SHAP feature importance refers to the sum of the absolute SHAP values across all samples, reflecting the overall contribution magnitude of a feature to the model’s prediction. The formula is

(16) $$FI_{i}=\frac{1}{N}\sum_{j=1}^{N}|\phi_{i}^{\left(j\right)}|$$

In this expression, $FI_{i}$ denotes the importance of feature i, and $\phi_{i}^{\left(j\right)}$ is the SHAP value of feature i in the j-th sample. The ranking of feature importance intuitively reflects the overall contribution level of each nonlinear parameter to the model’s classification decision.

The SHAP beeswarm plot summarizes the distribution of class-specific feature attributions across test segments. The horizontal axis shows each feature’s contribution to the raw score (margin) for the target class, here the ictal class S. Positive SHAP values increase the raw score for class S relative to the background expectation, whereas negative values decrease that score. Color encodes the z-standardized feature value used by the model: red denotes a relatively high standardized value and blue a relatively low standardized value. Each point represents one time-series segment. These attributions describe model behavior under the specified background and dependence assumptions and should not be interpreted as causal physiological effects.

The SHAP waterfall plot provides a local explanation for one test segment. Starting from the class-specific expected raw score, it shows how the standardized feature inputs additively move the raw margin toward the final model output for that segment. The displayed values are z scores rather than physical measurements. The plot improves the traceability of the fitted model output, but it does not identify abnormal physiological drift, establish clinical causality, or provide a standalone targeted diagnostic or treatment basis.

## 3. Results

Nine nonlinear dynamical features were first extracted from the dataset, followed by SHAP analysis to evaluate the contribution and interpretability of each feature.

### 3.1. Descriptive Waveform Overview

**Figure 1:**
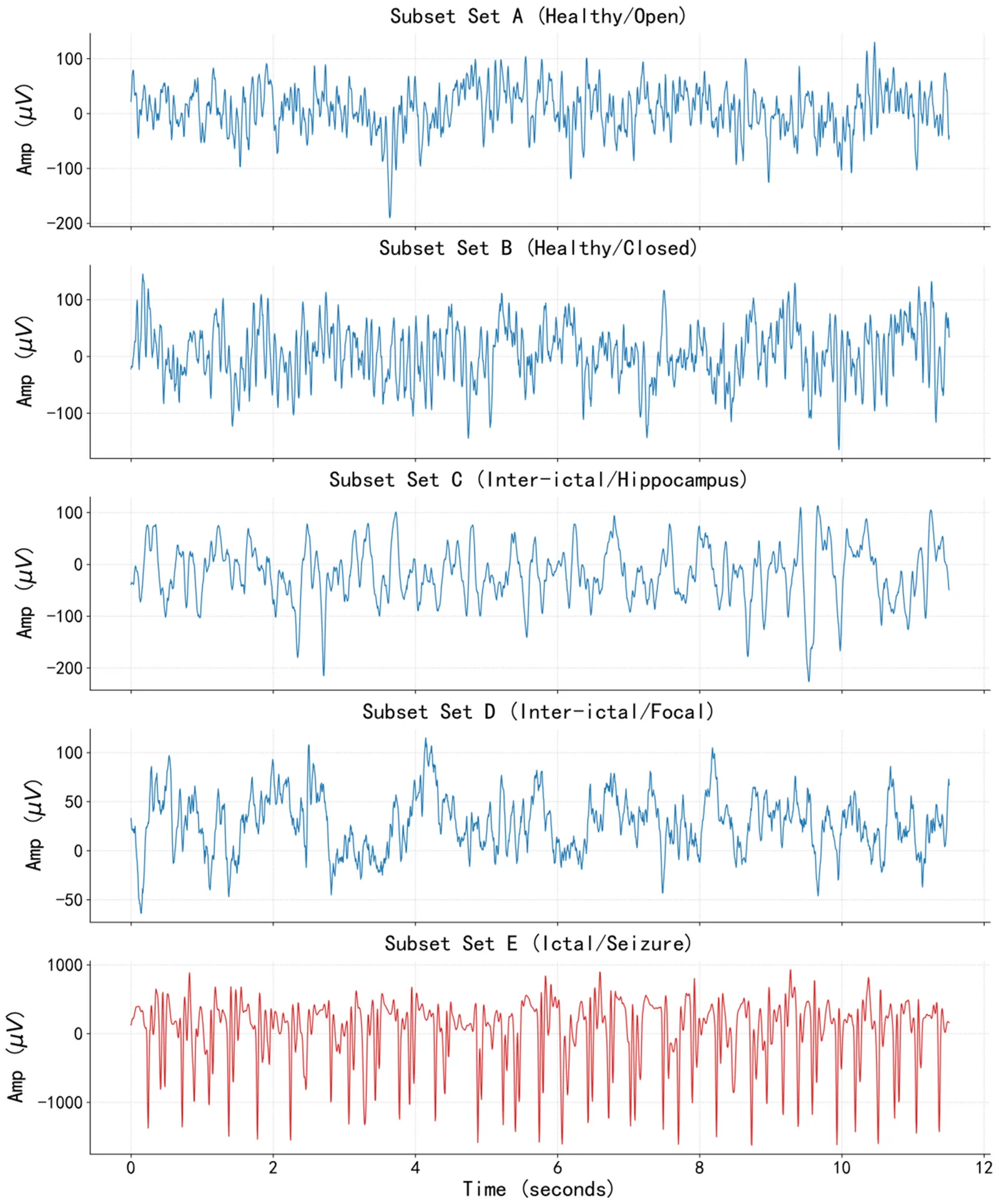
Representative waveform displays from the five EEG subsets. (Blue lines represent interictal EEG signals, and red lines represent ictal EEG signals).

The participant groups, recording locations, acquisition settings, and analysis workflow are specified in Section 2.1. Figure 1 provides representative waveform displays from the five public Bonn subsets before the duration-specific feature analysis. For descriptive presentation, Z and O represent healthy scalp recordings with eyes open and closed; N and F represent interictal intracranial recordings outside and within the epileptogenic region; and S represents ictal intracranial recordings. The three model classes were formed as Z/O, N/F, and S, as defined in Section 2.1. These group labels combine differences in subject population, recording location, and brain state.

From the perspective of waveform morphology: the normal control group (subsets Z and O) exhibits low-amplitude, desynchronized fast-wave activity; the interictal group (subsets N and F) occasionally shows spike-like discharges, predominantly with background rhythm; the ictal group (subset S) presents typical high-amplitude synchronized discharges, with continuous spike-and-slow-wave complexes, and the amplitude is higher than that of the other four groups. This result confirms the differences among different EEG states from a time-domain perspective, providing an intuitive data basis for subsequent nonlinear feature extraction.

### 3.2. Nonlinear Feature Extraction

From each EEG signal sample, nine core nonlinear parameters are extracted to construct a multidimensional nonlinear feature space. The extracted parameters include sample entropy, fuzzy entropy, permutation entropy, Rényi entropy, dispersion entropy, Hurst exponent, C0 complexity, Higuchi fractal dimension, and Lempel–Ziv complexity. These nine parameters systematically characterize the nonlinear dynamic characteristics of EEG signals from perspectives such as complexity and fractal properties.

To intuitively examine the distribution characteristics and discriminative ability of each nonlinear parameter across different categories of EEG signals, this study segments the signal with a 10 s window and violin plots of the nine core nonlinear parameters for three sample categories: normal state, interictal period, and ictal period.

**Figure 2:**
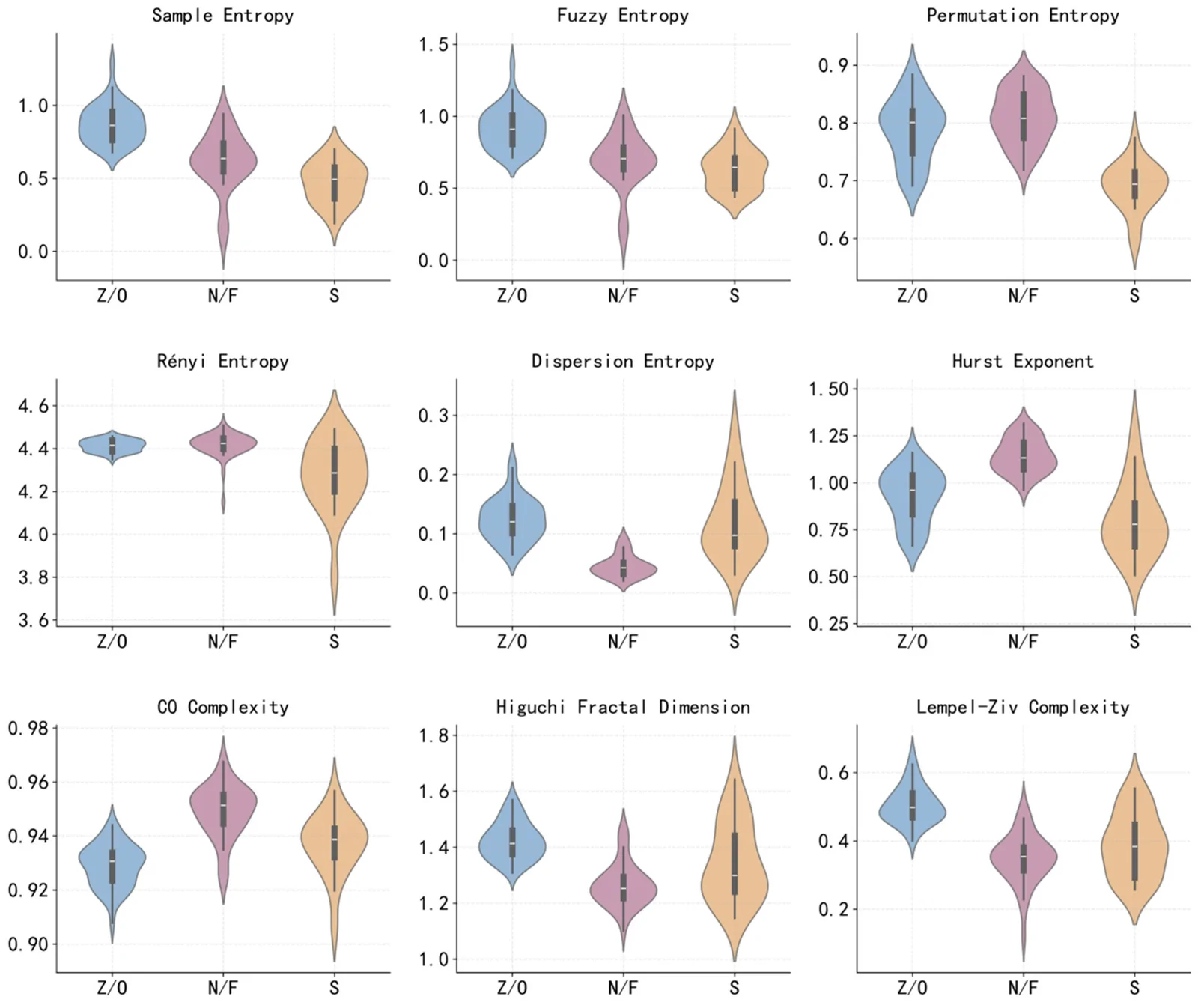
Distribution of the nine nonlinear parameters for the independently analyzed 10 s window condition.

Figure 2 shows that the nine nonlinear parameters exhibit differentiated central tendency and dispersion among the three sample classes. For several parameters, including permutation entropy, Renyi entropy, sample entropy, Hurst exponent, and Higuchi fractal dimension, the distributions show visible separation among groups. These descriptive patterns suggest that nonlinear features carry useful information for benchmark EEG state classification; however, visual separation alone does not replace inferential statistical testing, effect size estimation, or multiple-comparison correction.

### 3.3. Epileptic EEG Signal Classification Based on XGBoost

This experiment used the nine nonlinear parameters described above and trained the XGBoost classifier under the fixed recording-level split. For the 10 s condition, the test set contained 150 non-overlapping segments derived only from held-out original recordings.

**Figure 3:**
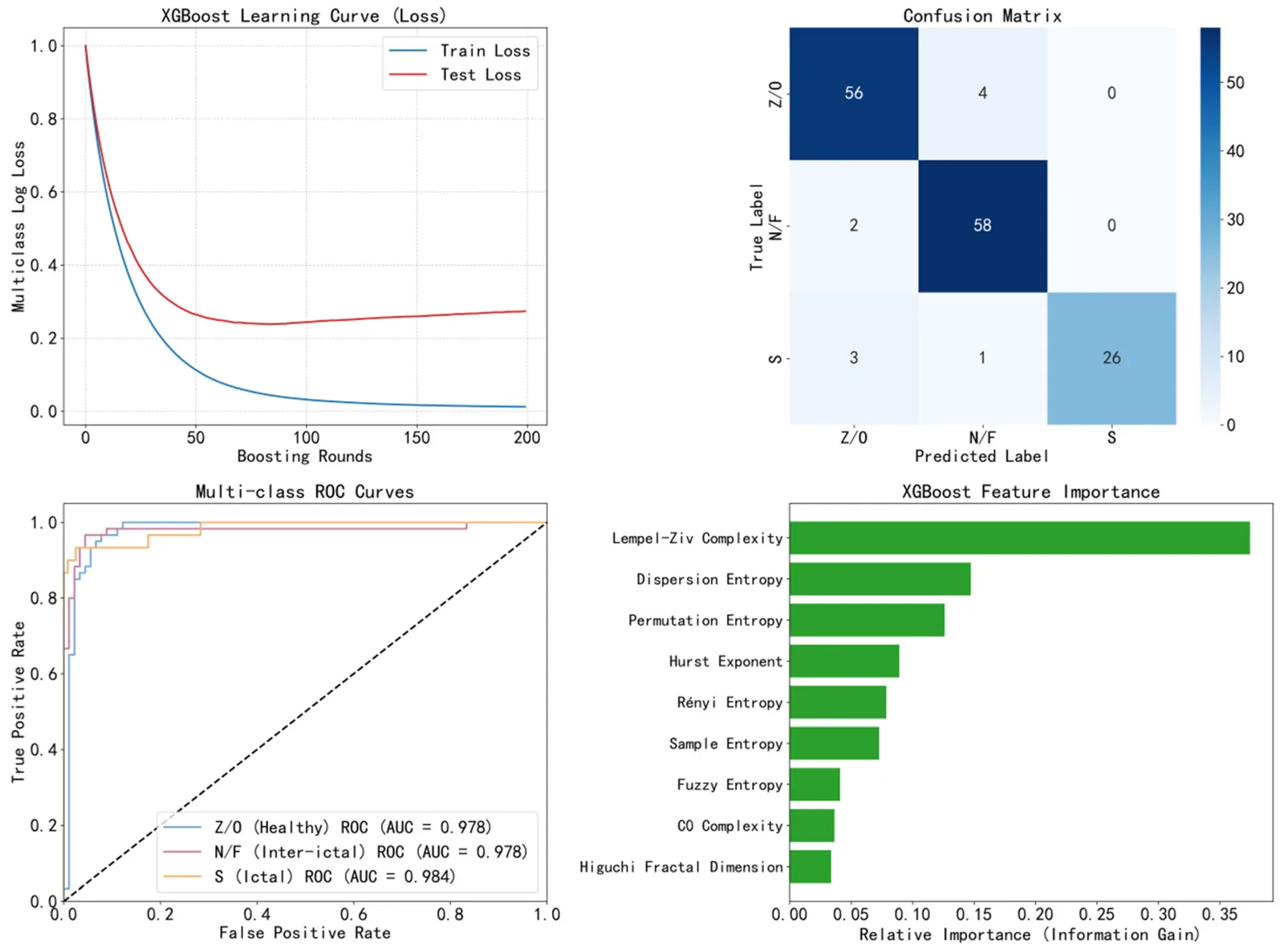
XGBoost classification results.

The fixed 10 s test run shown in Figure 3 produced 140 correct predictions among 150 test segments, corresponding to an overall accuracy of 93.3%. The class-specific AUC values shown in the ROC plot were 0.978 for Z/O, 0.978 for N/F, and 0.984 for S, yielding a macro-average AUC of 0.980.

From the perspective of feature contribution, LZC, dispersion entropy, and permutation entropy show relatively high contribution levels. However, in feature importance evaluation, XGBoost measures the purity improvement during the tree construction process. Its feature importance is based on the average reduction in the loss function when that feature is used for splitting across all decision tree nodes. However, XGBoost has two inherent drawbacks. The first is cardinality bias: if the data distribution of a feature is extremely continuous with a high cardinality consisting of many minute value differences, XGBoost’s splitting algorithm tends to perform multiple fine-grained splits on this feature, which leads to an artificial inflation of its gain. The second is inconsistency uncertainty: as the model changes, the importance of a given feature also varies, which violates the consistency principle. Therefore, in the following section, this paper further conducts SHAP-based interpretability analysis.

### 3.4. SHAP-Based Feature Interpretability Analysis

**Figure 4:**
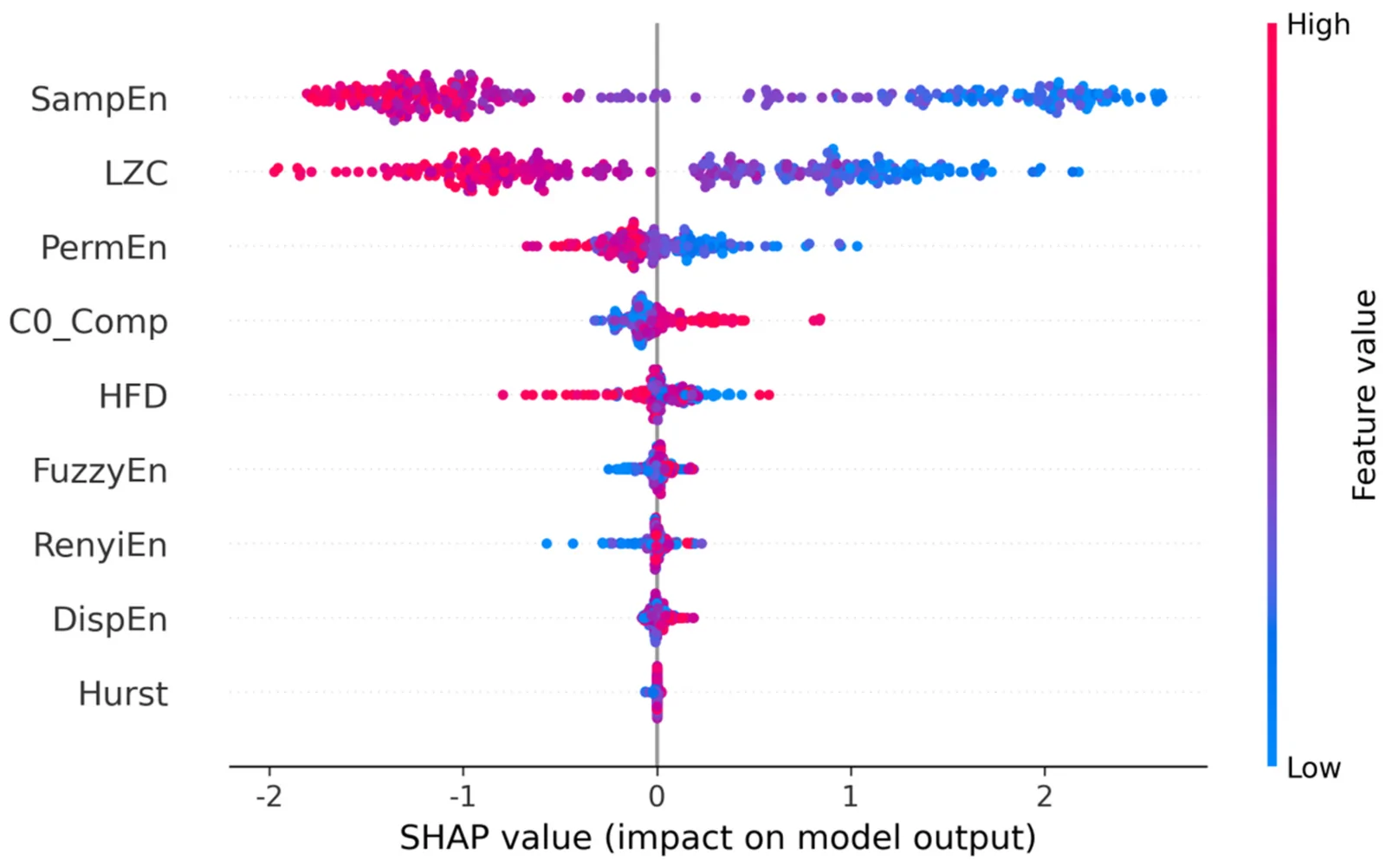
SHAP beeswarm plot for nonlinear indicators.

To further explore the importance of features, the experiment generated a beeswarm plot and a waterfall plot, as shown in Figure 4.

**Figure 5:**
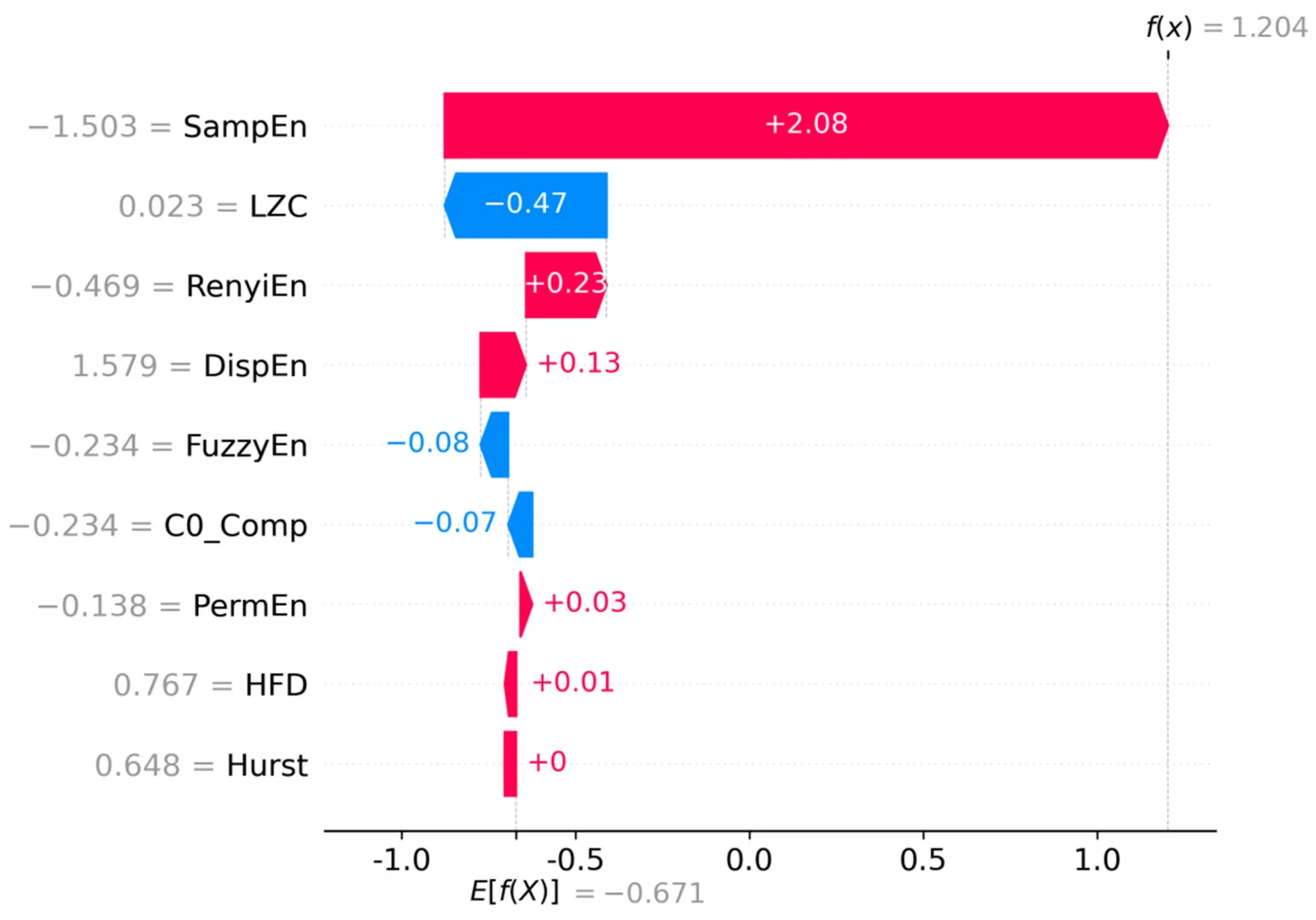
SHAP waterfall plot for nonlinear indicators. (Red denotes pushing for model fitting, and blue denotes restraining model fitting.).

For the ictal class, the beeswarm plot indicates that lower values of SampEn and LZC tend to push the fitted model, whereas higher values tend to push predictions away from that class. This pattern is consistent with reduced signal complexity during ictal segments in this benchmark dataset. These associations describe how the fitted model used standardized features in this split. They are not evidence that a high or low raw physiological measurement causes an ictal state. As shown in Figure 5.

As shown in the figure above, the SHAP ranking results indicate that SampEn and LZC are the dominant contributors to the fitted model output for the class. This result supports the usefulness of nonlinear complexity descriptors for benchmark EEG segment classification, while the clinical relevance and patient-level generalizability of these attributions require external validation.

### 3.5. Analysis of the Impact of Signal Duration on Classification Performance

The 1, 5, 10, and 20 s conditions were regenerated with the same feature definitions, split seeds, grouped train/validation/test procedure, hyperparameter grid, and evaluation metrics. Duration was treated as a descriptive sensitivity analysis rather than a prespecified model selection contest. Table 1 summarizes macro-level performance.

The 1 s condition had the lowest accuracy (0.910) and macro-sensitivity (0.908). At 5 s, accuracy was 0.953, macro-sensitivity was 0.949, and macro-AUC was 0.993. The 10 and 20 s conditions yielded accuracies of 0.933 and 0.956, macro-sensitivities of 0.922 and 0.951, and macro-AUCs of 0.980 and 0.994, respectively. Because these values were obtained from one fixed recording-level split without repeated grouped resampling or confidence intervals, the differences are descriptive only. They indicate lower performance at 1 s and broadly comparable point estimates from 5 to 20 s but do not establish a uniquely optimal window or support mechanistic claims about non-stationarity or state dilution.

Under the same fixed recording-level split, SVM had the highest descriptive point estimates for accuracy (0.975) and macro-AUC (0.996), while multinomial logistic regression achieved 0.961 accuracy, Random Forest 0.952, and XGBoost 0.933. Because repeated grouped resampling, confidence intervals, and formal paired comparisons were not performed, these single-split values do not establish predictive superiority of any classifier. XGBoost was retained as the primary explanatory model because it accommodates nonlinear effects and feature interactions and enables efficient model-specific attribution with TreeExplainer. Thus, the principal advantage of the XGBoost-SHAP framework in this study is the integration of nonlinear modeling with transparent, class-specific attribution rather than superior classification performance.

## 4. Discussion

The observed class-dependent nonlinear-feature distributions are qualitatively consistent with reports from Lu et al. [2,3,4] and Chen et al. [5], which also found entropy-, complexity-, and fractal-based representations useful for epileptic EEG classification. Tan et al. [6] similarly summarized the value of nonlinear measures across detection and prediction studies. Direct numerical comparison is not appropriate, however, because these laboratories used different class definitions, datasets, segmentation rules, validation units, and feature sets. The present result should therefore be read as an independent Bonn benchmark observation rather than evidence of superiority.

For the classifier and explanation components, Dweiri et al. [8] demonstrated the computational usefulness of XGBoost for single-channel seizure detection, while Rehman et al. [12], Dokare and Gupta [13], and Hasan et al. [14] combined machine learning with feature attribution methods in other EEG cohorts. The present contribution differs by evaluating nine nonlinear descriptors, four independent window duration conditions, and class-specific SHAP attribution within one recording-level-split pipeline. SHAP improves the transparency of the fitted model, but agreement with features reported by other laboratories remains associative and does not establish a shared physiological mechanism.

EEG was treated throughout as a stochastic, non-stationary process whose probability distribution can change over time. Nonlinear features are finite-sample statistical descriptors of that process, and their estimates may be less stable in very short windows. The 1 s condition was retained to examine this temporal–resolution trade-off and showed the lowest descriptive accuracy and macro-sensitivity under the present feature settings. Figure 2 displays the independently processed 10 s condition as a representative visualization, not because 10 s was demonstrated to be optimal. No 1 s and 10 s segments were mixed within one model.

Several design limitations remain. The Bonn subsets differ in subject group and recording region, and no independent external patient cohort was used. Although original recordings were split before segmentation, the present analysis reports one fixed split without repeated grouped cross-validation, confidence intervals, or formal statistical comparisons. Comparator models were included only as single-split descriptive benchmarks, and no feature–group ablation was performed; therefore, the independent incremental value of the entropy, fractal, and complexity feature groups cannot be determined from the current analysis. These constraints limit generalization and should be addressed using patient-level data, repeated grouped resampling, prespecified statistical tests, feature-group ablation, and external validation.

The current XGBoost/SHAP analysis is a research framework for benchmark pattern quantification and mechanism-oriented hypothesis generation, not a replacement for expert review. It does not evaluate continuous clinical EEG, prospective seizure monitoring, seizure prediction, or real-world diagnosis; clinical use would require patient-level validation, prospective testing, and assessment under realistic noise, artifact, and class-imbalance conditions.

## 5. Conclusions

This study evaluated whether nonlinear descriptors of stochastic and non-stationary EEG segments can support interpretable XGBoost classification on the Bonn benchmark. The authors’ contribution was the integrated, leakage-controlled pipeline combining nine nonlinear features, four independent window-duration conditions, multiclass XGBoost, and class-specific SHAP analysis. The principal conclusions are as follows.

(1) The nine extracted nonlinear parameters can characterize differences among Bonn benchmark EEG segment classes. Distributional analysis shows that dispersion entropy, Hurst exponent, and Higuchi fractal dimension exhibit visible group differences, indicating potential class-discriminative value under the present dataset.
(2) SHAP interpretability analysis clarifies the differentiated contributions of each nonlinear parameter to the fitted XGBoost model. In the S-class explanation, sample entropy and LZC are the most influential features, but the attribution results should be interpreted at the model level rather than as causal biomarkers.
(3) Signal duration affected the descriptive point estimates under the fixed, non-overlapping segmentation protocol. The 1 s condition had the lowest performance, whereas the 5, 10, and 20 s conditions were broadly comparable. Because no repeated grouped resampling or formal comparison was performed, these results do not establish a uniquely optimal duration.

In summary, the epileptic EEG segment recognition framework constructed in this study, based on multiple nonlinear dynamic features and a SHAP interpretable model, provides benchmark-level evidence for interpretable machine learning analysis of epileptic EEG signals.

## Figures and Tables

**Table 1:** Comparison of XGBoost model classification performance under different signal durations.

Segment Duration	Mean Accuracy	Macro-Precision	Macro-Specificity	Macro-Sensitivity	Macro-AUC
1 s	0.910	0.914	0.952	0.908	0.982
5 s	0.953	0.958	0.975	0.949	0.993
10 s	0.933	0.946	0.963	0.922	0.980
20 s	0.956	0.960	0.977	0.951	0.994

## Data Availability

The EEG dataset analyzed in this study is publicly available at https://www.ukbonn.de/epileptologie/arbeitsgruppen/ag-lehnertz-neurophysik/downloads/ (accessed on 10 March 2020). The source code supporting the findings of this study is available from the corresponding author upon reasonable request.

## Abbreviations

The following abbreviations are used in this manuscript:

EEG	Electroencephalogram
XGBoost	Extreme Gradient Boosting
SHAP	SHapley Additive exPlanations
SampEn	Sample entropy
DE	Dispersion entropy
LZC	Lempel–Ziv complexity
HE	Hurst exponent
